# How Cognitive Models of Human Body Experience Might Push Robotics

**DOI:** 10.3389/fnbot.2019.00014

**Published:** 2019-04-11

**Authors:** Tim Schürmann, Betty Jo Mohler, Jan Peters, Philipp Beckerle

**Affiliations:** ^1^Work and Engineering Psychology Research Group, Human Sciences, Technische Universität Darmstadt, Darmstadt, Germany; ^2^Amazon, Tübingen, Germany; ^3^Intelligent Autonomous Systems Group, Department of Computer Science, Technische Universität Darmstadt, Darmstadt, Germany; ^4^Max Planck Institute for Intelligent Systems, Tübingen, Germany; ^5^Elastic Lightweight Robotics, Department of Electrical Engineering and Information Technology, Robotics Research Institute, Technische Universität Dortmund, Dortmund, Germany; ^6^Institute for Mechatronic Systems, Mechanical Engineering, Technische Universität Darmstadt, Darmstadt, Germany

**Keywords:** cognitive models, human body experience, multisensory integration, robotics, assistive devices, humanoids

## Abstract

In the last decades, cognitive models of multisensory integration in human beings have been developed and applied to model human body experience. Recent research indicates that Bayesian and connectionist models might push developments in various branches of robotics: assistive robotic devices might adapt to their human users aiming at increased device embodiment, e.g., in prosthetics, and humanoid robots could be endowed with human-like capabilities regarding their surrounding space, e.g., by keeping safe or socially appropriate distances to other agents. In this perspective paper, we review cognitive models that aim to approximate the process of human sensorimotor behavior generation, discuss their challenges and potentials in robotics, and give an overview of existing approaches. While model accuracy is still subject to improvement, human-inspired cognitive models support the understanding of how the modulating factors of human body experience are blended. Implementing the resulting insights in adaptive and learning control algorithms could help to taylor assistive devices to their user's individual body experience. Humanoid robots who develop their own body schema could consider this body knowledge in control and learn to optimize their physical interaction with humans and their environment. Cognitive body experience models should be improved in accuracy and online capabilities to achieve these ambitious goals, which would foster human-centered directions in various fields of robotics.

## 1. Introduction

Multisensory integration is a key cognitive function for human body experience (Giummarra et al., 2008; Christ and Reiner, 2014) and cognitive modeling research suggests that it is performed in a Bayesian manner (Deneve and Pouget, 2004; Körding et al., 2007; Orbán and Wolpert, 2011; Clark, 2013). Sun (2008) defines cognitive models as computational models relating to one or multiple cognitive domains or functionalities. While this model class is occasionally referred to as computational models, the authors rely on the term “cognitive models” to reduce ambiguity with relation to Marr (1982) computational level of analysis, to which cognitive models do not need to be limited to. Cognitive models of the aforementioned integration processes consider sensorimotor precision with respect to the corresponding individual modalities (Berniker and Körding, 2011) and can determine posterior estimates based on prior knowledge and sensory information.

From the authors' perspective, modeling, and simulating multisensory integration mathematically could potentially help to endow (humanoid) robots with more human-like capabilities and improve scenarios with tight physical human-robot interaction, e.g., in assistive devices. The increased interest and progress made toward such capabilities has stimulated research in this direction from which we can draw on a variety of works on robotic self-perception (Sturm et al., 2009; Ulbrich et al., 2009; Lanillos et al., 2017; Lanillos and Cheng, 2018), reviews analyzing connections between human body experience and robotics (Hoffmann et al., 2010; Schillaci et al., 2016; Beckerle et al., 2017) as well as recent works that propose cognitive models of bodily illusions using Bayesian approaches (Samad et al., 2015). Such illusions rely on targeted modulations of multisensory stimulation and make participants perceive artificial limbs as their own (Botvinick and Cohen, 1998; Giummarra et al., 2008; Christ and Reiner, 2014).

Obviously, such effects are of utmost interest for assistive robotics since exploiting them by means of control could help to integrate such devices into their user's body schema (Ehrsson et al., 2008; Christ and Reiner, 2014; Beckerle et al., 2017). Moreover, the body schema is directly connected to the sense of agency (Longo et al., 2008; Kannape et al., 2010), i.e., the feeling to have control over the own body. In assistive robotics, it is important to account for changes in each user's body schema to foster their sense of agency. Meanwhile, endowing humanoids with a body schema is promising for control reasons, e.g., keeping safe distances or reaching for targets (Roncone et al., 2015, 2016). As a psychological concept, the body schema can be understood as an adaptable (Somogyi et al., 2018), subconscious representation of the body's characteristics (Gallagher and Cole, 1995; Mayer et al., 2008), e.g., its kinematics and dynamics, which makes it promising for hand/tool-eye coordination in humanoid robots (Ulbrich et al., 2009). Psychological studies suggest that the representations of the human body itself and the representation of the environment in reach, i.e., the peripersonal space, are closely linked (Serino et al., 2007; Cléry and Ben Hamed, 2018). This appears to enable a flexible discrimination between the self and the environment including adaptation when using tools (Holmes and Spence, 2004; Hoffmann et al., 2010), a capability that is rather underdeveloped in contemporary humanoid robots (Hoffmann et al., 2010). Therefore, cognitive models that go beyond models which described the kinematic structure or dynamic properties of a robot as reviewed in Nguyen-Tuong and Peters (2011), seem to be required.

## 2. Cognitive Models

Among the existing cognitive models, we assume Bayesian and connectionist approaches to be most suitable for achieving human-like body representations in robots. In this section, we detail how we arrive at this assumption by considering conceptual foundations and empirical applications of the modeling approaches. An interesting example for their application are bodily illusion experiments, where the distance between the perceived position of the real limb and its indicated position, i.e., the proprioceptive drift, is understood as an objective, but also debated, measure of embodiment (Giummarra et al., 2008; Pazzaglia and Molinari, 2016). The assumption that participants could fuse multisensory information in a Bayesian process (Berniker and Körding, 2011) motivated the development of computational models that aim to estimate the proprioceptive drift from empirical input data (Samad et al., 2015). Accordingly, these Bayesian cognitive models compute estimations of the proprioceptive drift (Samad et al., 2015) and thereby propose quantitative approximations to the generative process of human sensorimotor integration. However, these models exhibit limited estimation accuracy and are constrained to offline application to the experimental population as a whole (Samad et al., 2015).

Marr (1982) defines three general levels of analysis for cognitive models: the computational, algorithmic, and implementational levels. The aforementioned research describing Bayesian cognitive models of multisensory information (Berniker and Körding, 2011; Samad et al., 2015) tends to define these inferential problems on the computational level. Here, modelers define the logic and structure of a computational problem. Yet, cognitive models of human body experience might also benefit from extension to deeper modeling levels (Griffiths et al., 2012), e.g., the algorithmic level, defining the processes and representations involved in solving the computational problem. Combined model specifications on the computational and algorithmic level can foster the prediction and explanation of seemingly error-prone or paradoxical behavior, as observed in research on causal reasoning (Tenenbaum et al., 2007) or decision making (Srivastava and Vul, 2015).

As a separate school of thought, connectionism commonly employs artificial neural networks to represent information in patterns of activation. While artificial neural networks do not need to be implemented in a neurally plausibile way by human standards, connectionism is historically inspired by the idea of creating “brain-like” systems (Thomas and McClelland, 2008). This aspect ties connectionist models to the implementational level of analysis (Marr, 1982), which concerns the physical realization of a model's computation in biological or technological hardware. Similarly to Bayesian approaches, multisensory integration can be approached in a connectionist fashion (Quinlan, 2003; Zhong, 2015). In fact, interpreting the weights of an artificial neural network as conditional probability relations creates a strong similarity between connectionist and Bayesian models of cognition (Thomas and McClelland, 2008). If a connectionist implementation mimics the close-to-optimal sensorimotor integration that humans seem to perform (Körding and Wolpert, 2006), its prediction of body experience should thus be alike Bayesian estimations.

While there are other schools of cognitive modeling (Sun, 2008), we focus on Bayesian approaches due to their relation to human sensorimotor behavior (Körding and Wolpert, 2006; Franklin and Wolpert, 2011) and connectionism because of its relation to developmental psychology (Shultz and Sirois, 2008) and developmental robotics (Lungarella et al., 2003). Being conceptually similar, both approaches can either be used to investigate the generative process behind human sensorimotor behavior or to control sensorimotor capacities in artificial systems. Yet, connectionism appears to be employed mostly without a direct relation to human performance (Katić and Vukobratović, 2003; Metta et al., 2010, 2017; Pasquale et al., 2015; Lakomkin et al., 2018), although some examples draw commendable design references from human neurobiology (Morse et al., 2010).

## 3. Applications in Robotics

We expect that cognitive models of human body experience will improve the capabilities of robotic systems and discuss potentials and challenges of their implementation and utilization. Specifically, assistive robotic devices and humanoid robots are taken as examples that highlight the possibilities and their prospective effects.

Hoffmann et al. state that robots, which could include humanoids and assistive devices, need two things to perform a goal-directed action: a certain knowledge about their physical self and the mapping between their sensory and motor modalities (Hoffmann et al., 2010). In their review, they distinguish different kinds of kinematic body representations that are either fixed, self-calibrate to geometry changes, or are generated automatically, while only specific body representation models comprise dynamics (Hoffmann et al., 2010). In contrast to these explicit models, they describe implicit ones that represent the sensorimotor mappings, self-recognition, and temporal effects (Hoffmann et al., 2010). A more recent review by Schillaci et al. (2016) describes how explorative behaviors could drive motor and cognitive developments. Schillaci et al. describe such behaviors as a very ingenious method to acquire and maintain internal body representations in artificial agents, e.g., through MOdular Selection And Identification for Control (MOSAIC) models (Haruno et al., 2001).

### 3.1. Assistive Devices

Achieving a seamless integration of assistive robotic devices in supporting users' movements requires a better understanding of both human body schema integration and knowledge representation about the users' motor capabilities. A crucial point is to avoid excessive device activity, which might hinder body schema integration due to being perceived as external activity. By establishing the underlying processes of multisensory integration as elements of cognitive models, we propose that effects of robotic assistance can be predicted in multiple movement scenarios. These predictions can be used to adjust sensory feedback to the user by comparing estimated and required forces and torques to solve motor tasks over time. In case of a mismatch between actual and desired value, the need for changing motor behavior might be communicated to the user through (modulated) sensory feedback, which could also be used to foster co-adaptation of user and device (Beckerle et al., 2017, 2018).

Hence, such models could facilitate user- and application-specific assistance to assist-as-needed by the individual and in different situations. We argue that online models of required users' motor activities could help to complement and adjust assistance, easing both habituating to and weaning from it.

While assistance-as-needed might also be implemented through inverse dynamics models, cognitive models could help to tune factors that modulate the user's body experience. Human-in-the-loop experiments, e.g., robot-aided bodily illusions, could help to reveal those factors and how they influence embodiment (Beckerle et al., 2018). With this knowledge, not only force/torque or motion control, but also human-machine interfaces could be optimized with respect to embodiment of the assistive device, e.g., providing appropriate tactile feedback to shape the representation of the artificial limb (Giummarra et al., 2008; Beckerle et al., 2017). Through in-depth knowledge of the human cognitive body representation and a corresponding model-based control of the assistive device, co-adaptation might be systemized to achieve a congruent representation. Additionally, improper operation of the device by the user might be anticipated automatically and compensated for by means of control. While representing a great potential, the vision of assistive devices that understand their user's body experience and adapt to it—individually and online—also outlines the requirement for radical improvements of contemporary models.

### 3.2. Humanoid Robots

While assistive devices should interact seamlessly with their users, humanoid robots are intended to autonomously behave in a human-like manner. We expect that endowing humanoid robots with their own body schema and peripersonal space could tackle various recent issues. For instance, humanoids that have an understanding of their physical properties and environment could adapt their behavior to humans and the environment during physical, cognitive, and social interaction. Consider the example of standing in a crowded elevator: humans would adapt their relative positions, i.e., keep certain distances to others, while contemporary humanoid robots might not. The relation between knowledge about one's own body, obstacle avoidance, and social norms in interacting with humans highlights the potential of providing humanoid robots with a sense of their body and its environment.

While humanoid robots might be expected to produce human-like behavior regardless of the behavior generation process, this process itself might be required to be human-like. Developmental robotics research draws its appeal at the edges of engineering, developmental psychology, and cognitive science by potentially improving the capabilities and autonomy of robots. Moreover, it promises to simultaneously reveal how developmental models may perform when implemented in a robotic body (Lungarella et al., 2003; Asada et al., 2009). Recent research enables humanoid robots to develop several forms of body representation (Martinez-Cantin et al., 2009; Lara et al., 2016; Hoffmann et al., 2018) or learn movement generation (Metta et al., 2017). While achieving flexible, autonomous behaviors, most contemporary studies do communicate about the human-likeness of the behavior generation, but lack a formal evaluation method comparing it to human behavior.

**Figure 1:**
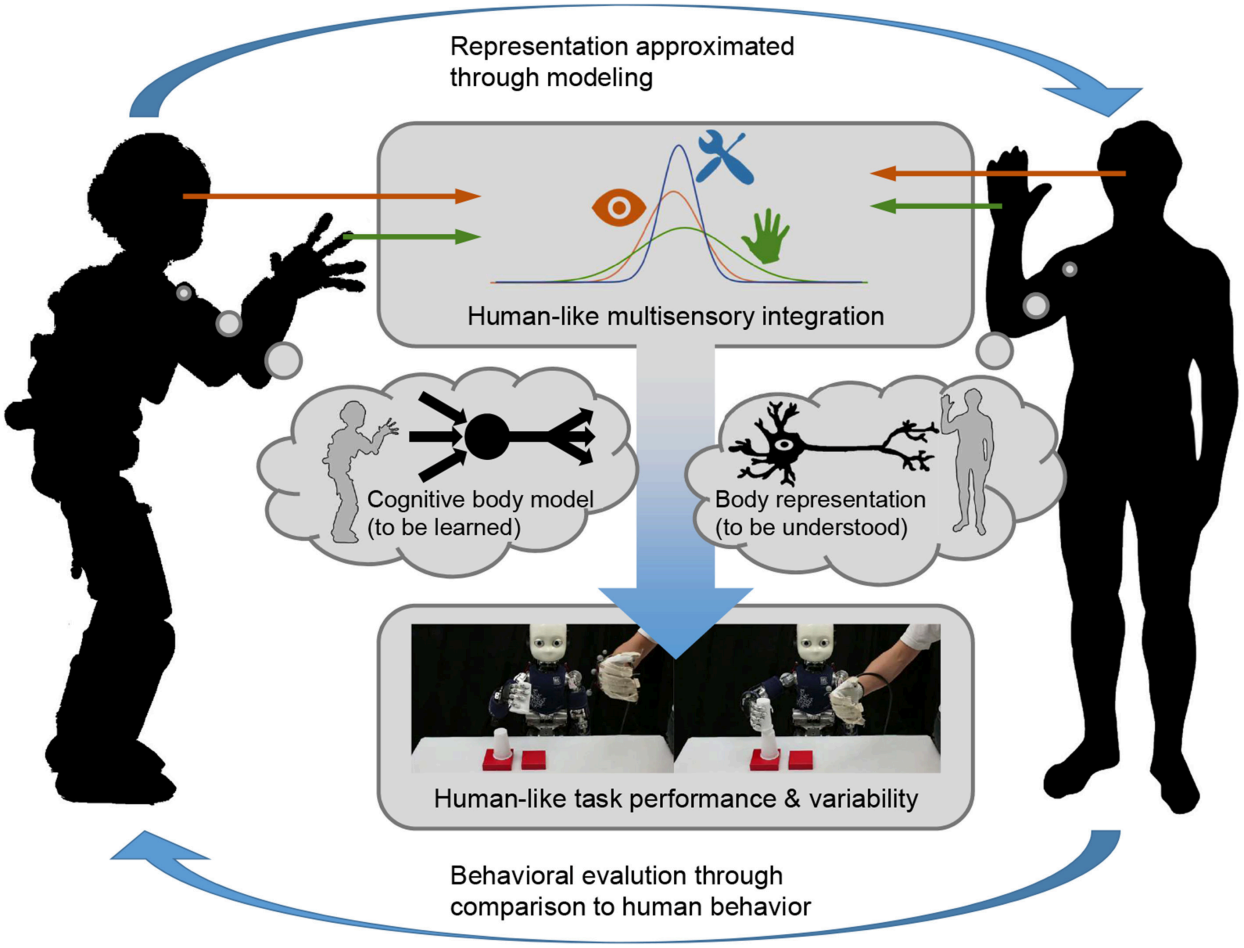
Control adaptation through cognitive models of human body experience during sensorimotor manipulation: multisensory data from human/robot perception is processed by a cognitive model. Using it for robot control, a human-like body representation is developed and, finally, human-like behavior generation is reached. In an iterative process, human cognitive function might be researched fundamentally and, in turn, models could be advanced through behavioral evaluation based on human data.

Although these methods may be sufficient to improve autonomous behavior, we suspect differences between the robotic and human behavior generation processes. Specifically, these differences may show when observed human performance exhibits a variability that is not strictly required by the kinematic or dynamic properties of the task at hand. We hypothesize that complementing established kinematics and dynamics models through psychologically motivated cognitive models will help to approach a human-like behavior generation process and improve the design of behaviors and interactions in robots. While we believe that both Bayesian and connectionist modeling approaches could be employed for this, a comparison to actual human behavior is mandatory for evaluation. An appropriate example might be the sensorimotor task presented in Körding and Wolpert (2004): participants were asked to point at a target in virtual reality while their cursor underwent a lateral shift relative to the actual location their finger controlling it. In this human experiment, Krding and Wolpert conclude that participants internally represented the statistical properties of the task manipulation in consistency with Bayesian inference. Exposing a humanoid robot to a comparable task, three stages might finally lead to human-like performance. Firstly, precise sensors could measure the lateral shift to enable the robot to execute a corrected trajectory. Secondly, a more human-like behavioral variability might be reached by artificially restricting the corrected trajectory through an arbitrary error term. Finally, we postulate that control adaptation through cognitive models could intrinsically yield fully human-like behavior generation and might result in similar observations as those found by Körding and Wolpert (2004). Figure 1 sketches how this might be implemented for the example of multisensory integration during sensorimotor manipulation, which applies to assistive devices similarly.

Pioneering work shows how the iCub robot can learn a peripersonal space model from data acquired via a whole-body artificial skin and physical contact with the environment (Roncone et al., 2015, 2016). While this approach is still rather engineered and does not try to approximate human behavior generation, it achieves sampling rates that enable online combination with control and is capable to predict contacts between the whole body of the robot and its environment. This information is used to design a controller that can either implement a safety margin around the body of the robot or support reaching objects in the robot's vicinity (Roncone et al., 2015, 2016).

## 4. Conclusion

Current developments of cognitive body models, Bayesian as well as connectionist ones, have the potential to push assistive robotic devices by making them understand their users' body experience and humanoid robots by endowing them with own body knowledge. Assistive devices might utilize this knowledge by adaptive control improving their integration into their users' body schemes, i.e., devices could foster their embodiment themselves. Further, we postulate that such models might give humanoid robots a feeling for their own body and its surrounding that can be qualitatively comparable to human body perception, should the situation demand it. In both cases, we deem machine learning to be very helpful: assistive devices might learn how to improve their embodiment user-specifically, while humanoid robots could not only model their environment, but also improve their motions based on extensive body knowledge.

Future research should therefore improve models with respect to accuracy, specifications for individual users, and online capabilities. Therefore, experiments to determine modulating factors as well as prior knowledge about sensory precision should be improved, e.g., by human-in-the-loop approaches. A next step might be an integration of cognitive models with higher-level self-perception architectures as proposed by Lanillos et al. (2017), Asada et al. (2009), and Morse et al. (2010) and their application for purposes of control (Roncone et al., 2015, 2016) or hand/tool-eye coordination (Ulbrich et al., 2009). Therefore, the discussed cognitive models might be combined with established kinematic or dynamic models, which could be driven by model learning of an integrated body representation (Haruno et al., 2001; Nguyen-Tuong and Peters, 2011; Schillaci et al., 2016). Thereby, humanoids and assistive devices might be provided with more human-like behavior and improved capabilities to interact with human partners.

## Author Contributions

TS and PB coordinated the development of the paper and the integration of individual contributions. BM and JP contributed content, opinions, and references. All authors revised the manuscript. All work for BM was done at TU Darmstadt before she began working at Amazon.

### Conflict of Interest Statement

BM is employed by Amazon, Tübingen, Germany. The remaining authors declare that the research was conducted in the absence of any commercial or financial relationships that could be construed as a potential conflict of interest.
